# Body Adiposity Index: Its Relevance and Validity in Assessing Body Fatness of Adults

**DOI:** 10.1155/2014/243294

**Published:** 2014-01-22

**Authors:** Shilpi Gupta, Satwanti Kapoor

**Affiliations:** Obesity Research Unit, Physiological Anthropology Laboratory, Department of Anthropology, University of Delhi, New Delhi 110007, India

## Abstract

*Background*. One of the limitations of body mass index is its accuracy to assess body fatness. To address this limitation, a new index, body adiposity index, has been developed. However its validity needs to ascertained. *Objective*. Our aim was to investigate sex-specific relationship between BAI, BMI, and percent body fat in an endogamous population of Delhi, India. *Method*. Data was collected from 578 adults on bodyweight, height, skinfold thicknesses, hip circumference, waist circumference, and systolic and diastolic blood pressure. Pearson's correlations were calculated for BAI and BMI with PBF. Differences in the correlation coefficients were examined using Fisher's *z* tests. Receiver operating characteristic analysis was used to compare the predictive validity and to determine optimal cut-off values. Odds ratios were calculated to assess the risk of having hypertension using the proposed cut-off points. *Results*. The correlations of PBF with BMI (men: *r* = 0.83; women: *r* = 0.71) were stronger than those with BAI (men: *r* = 0.66; women: *r* = 0.58). In men, the sensitivity and specificity of BAI to predict hypertension were higher than other anthropometric markers but lower than BMI. In women, the sensitivity of BAI was higher than BMI and WC. *Conclusions*. BAI can be used as an additional marker for screening population; however its validity needs to be demonstrated on other populations too.

## 1. Introduction

The body mass index (BMI) is a heuristic proxy for human body fat based on an individual's weight and height. It was invented by Adolphe Quetelet between 1830 and 1850 [1]; since then it has been used to assess body fat. BMI is a fascinating anthropometric index because it meets the first four requirements for an ideal method, that is, (1) initial cost, (2) training of the operator, (3) maintenance and operating costs, and (4) precision [2]. The two instruments (scale and anthropometer) that are required are inexpensive, require minimal training to use, and are virtually maintenance-free; repeat values can be obtained with good precision and with no fear of exposure hazard. It has been shown to correlate significantly with other measures of adiposity too [3].

A graded classification of overweight and obesity using BMI classification provides valuable information about increasing body fatness. It allows meaningful inter- and intrapopulation comparison of body weight and identifies individuals or groups at risk of morbidity and mortality, thus paving the way for the identification of priorities in intervention at an individual or community level and for evaluating the effectiveness of such interventions. However, the accuracy of BMI in assessing the body fatness of an individual remains debated. Although BMI has been widely used as a surrogate measure of adiposity, it is a measure of excess weight relative to height, rather than excess body fat. It does not distinguish between fat mass and lean body mass. Besides the distribution of fat over the body is not captured. There is accumulating evidence that excess visceral fat increases the risk of chronic metabolic disorders such as cardiovascular disease and stroke [4]. Thus other adiposity measures like waist circumference, waist-to-hip ratio (WHR), and skinfold thickness supplement information regarding body fatness in addition to BMI.

Recently, Bergman et al. [5] have introduced a new index of adiposity, namely, body adiposity index (BAI), to counter the limitation of BMI. They reported stronger correlations of BAI with percentage body fat (PBF) determined by dual-energy X-ray absorptiometry (DEXA) than that of BMI. Although BAI was reported as a better predictor of PBF, however, certain questions need to be addressed before accepting it as a new measure of body adiposity. The index was developed in samples of Mexican-American and black individuals. It needs to be validated on other populations too. Further sexual dimorphism has not been taken into account. As mentioned by Schulze and Stefan [6], cut-offs of body fat in relation to type 2 diabetes, cardiovascular morbidity, and mortality need to be constructed.

In the present study, the validity of BAI has been investigated in North Indian population. In addition, the sex-specific relationship between BAI and PBF was examined. Cut-off of PBF estimated by BAI was also constructed. Furthermore, odds of hypertension were also computed with the derived cut-offs of BAI.

## 2. Materials and Methods

### 2.1. Subjects

Cross-sectional study was conducted on Aggarwal Baniyas, an endogamous caste group of Delhi. Data was collected during 2008-2009 by a household survey on 578 adult Aggarwal Baniyas (271 men, 307 women) aged 30 years upwards. Nonresponse rate for all the variables of interest was 2.6% for men and 4.9% for women in all cases. Final analysis was performed on the data of 264 men and 292 women with complete information. Information was collected during household visits using a schedule that included information on body weight, height, skinfold thicknesses, hip circumference, waist circumference, and systolic and diastolic blood pressure. Written informed consent was obtained from all participants and the protocol of the study was approved by the ethical committee of the Department of Anthropology, Faculty of Sciences, University of Delhi, India.

### 2.2. Measurements

Body weight was measured by using spring balance to the nearest 500 g, stature with the help of anthropometer to the nearest mm. Waist circumference and hip circumference were measured with a nonstretchable steel tape to the nearest 0.1 cm. The anthropometric measurements were taken according to the standardized techniques described by Weiner and Lourie [7]. Measurements were conducted by trained personnel and all instruments were calibrated once weekly. As an indicator of fatness, body mass index was calculated as weight in kg divided by stature in meter square. BAI was calculated as hip circumference in centimeters divided by height in meters to the 1.5 power minus 18. The biceps, triceps, suprailiac and subscapular skinfold thicknesses of the subjects were measured with Holtain skinfold calipers which exerted a constant pressure of 10 gm/mm^2^ over the contact surface. Published equations of Durnin and Womersley [8] were used to calculate BF% using skinfolds.

Blood pressure (BP) was measured using manual (mercury) sphygmomanometer. The subject was asked to sit relaxed on a chair with her/his arm supported comfortably at the vertical level. The pressure cuff was applied closely to the upper arm; the cuff was rapidly inflated until the artery was completely occluded. The stethoscope was then placed lightly over the brachial artery and the mercury column was allowed to fall at the rate of 2 mmHg per second. The systolic blood pressure was determined by the appearance of Korotkoff sound. After recording the systolic blood pressure, the mercury column was allowed to fall further till the sound ceased to be tapping in quality, became fully muffled, and finally disappeared. The level where it disappeared was taken as diastolic blood pressure. Three consecutive readings were taken for each measurement and their mean was taken as the final reading.

### 2.3. Statistical Analysis

Results have been described by summary statistics such as mean and standard deviation. Sex-specific partial Pearson correlations were calculated for BAI and BMI with PBF separately. For total percentage body fat, differences in the correlation coefficients for BAI versus BMI were examined using *z*-tests after applying Fisher *z*-transformation [9, 10]. Receiver operating characteristic (ROC) analysis was used to compare the predictive validity and to determine optimal cut-off values [11–13]. Area under the curve (AUC) was also measured to determine the diagnostic power of a test and to describe the probability that a test would correctly identify subjects with the disorder. Optimal cut-off values of BAI were measured by calculating the sensitivity and specificity of the anthropometric measurements at various cut-off points of BMI.

The accuracy of BAI, BMI, WC, and WHtR in detecting hypertension was examined by calculating sensitivity, specificity, and positive and negative predictive values (PPVs and NPVs) [14]. Odds ratios were calculated to assess the risk of having hypertension using the proposed cut-off points.

## 3. Results

The basic characters of our study have been published previously [15]. In brief, the mean age of males was 43.4 yr (SD ± 5.3) and that of females was 38.7 yr (SD ± 4.9). The prevalence of obesity was 42.7% in women and 21.2% in men. The prevalence of prehypertension was 48.7% and 45.6% among males and females, respectively. The percentage of hypertensive males and females was 21.6% and 7.4%, respectively.

**Figure 1:**
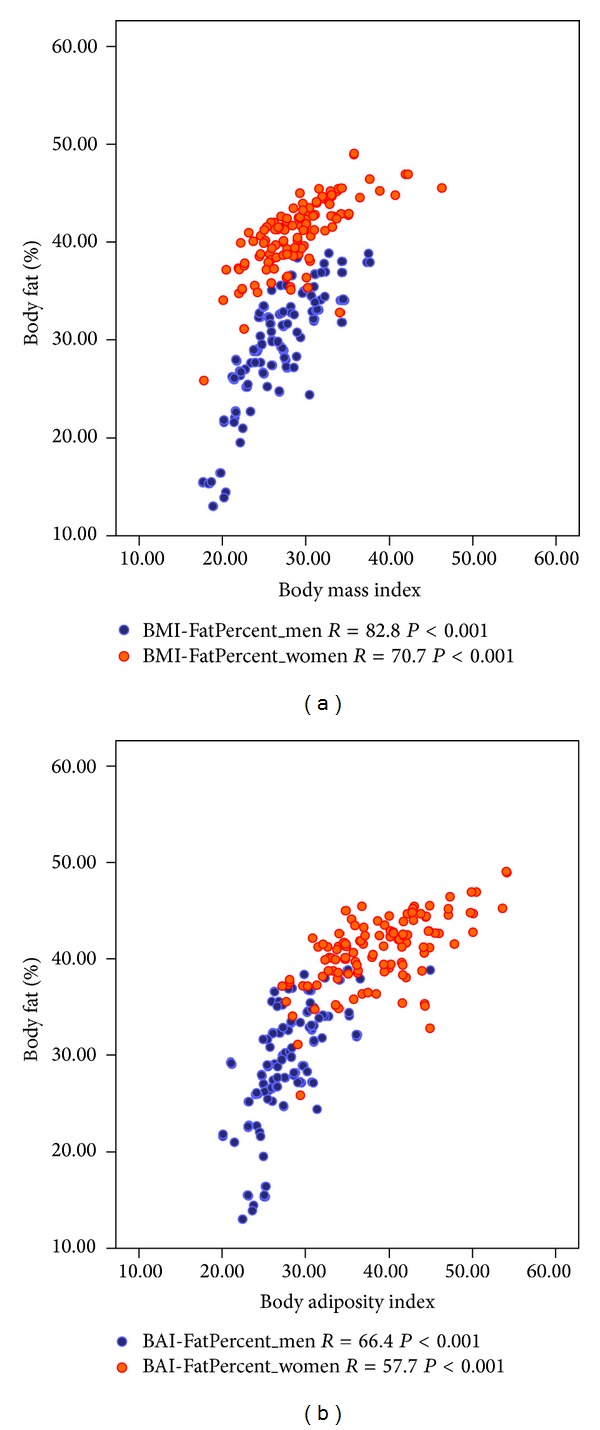
Relationship between percentage body fat versus body adiposity index and body Mass index. The lines indicate the linear relationships between the 2 variables.

While the correlations of BMI and BAI with PBF in both men (BMI: *r* = 0.83; BAI: *r* = 0.66) and women (BMI: *r* = 0.71; BAI: *r* = 0.58) were statistically significant, the correlations of PBF with BMI (men: *r* = 0.83; women: *r* = 0.71) were stronger than those with BAI (men: *r* = 0.66; women: *r* = 0.58) among both men and women (Table 1). The correlations between anthropometric indices and PBF were consistently lower for women compared with men. However when both sexes were taken together, the correlations of BAI with percentage body fat (0.80, *P* < 0.001) were better than BMI (0.70, *P* < 0.001). The regression model for BAI explained 66.4% of the variance in fat percentage in men and 57.7% in women, whereas the corresponding regression model for BMI explained 82.8% variance in men and 70.7% in women (Figure 1).

ROC curve analysis of BAI with BMI and BP is summarized in Table 2. The results of the AUC analysis were significantly different from what was expected by chance (*P* < 0.001). The values of AUC ranged from 0.90 to 0.94 for BMI while for BP they were 0.62 in women and 0.70 in men. The cut-off points of BAI were determined as the point of intersection of the curves for sensitivity and specificity. The BAI cut-off values corresponding to WHO-based BMI's cut-off were 27.2 in men and 36.4 in women, whereas BAI cut-offs based on derived population-based BMI cut-off values were 25.6 in men and 37.7 in women. BAI cut-offs based on BP risk were 25.3 in men and 37.7 in women.

The sensitivity and specificity of BAI to predict hypertension are presented in Table 3. Sensitivity and specificity of various anthropometric markers, that is, BMI, WC, WHR, and WHtR to predict hypertension have already been published [16]. In men, the sensitivity and specificity of BAI were higher than WC, WHR, and WHtR but lower than BMI. In women, the sensitivity of BAI was higher than BMI and WC.

Logistic regression analysis was performed to see if the determined cut-off points of BAI independently reflected the increased risk of having hypertension (Table 4). Odds of systolic and diastolic blood pressure on BAI were significant in both men and women; however, the odds were found to be higher in males than in females. The logistic regression analysis of other anthropometric markers for hypertension by optimal cut-offs has already been published [16]. In men, odds of hypertension on BAI were lower than other anthropometric markers while in women, they were higher for other anthropometric markers.

## 4. Discussion

We examined the validity of BAI to reflect PBF for adult men and women as compared to other anthropometric markers in an endogamous population of North India. Further sex-specific cut-off of BAI was determined based on BMI as well as BP.

In the present study, body fat appears to be more correlated with BMI than with BAI. The regression model including BMI explained greater variance in fat percentage as compared to BAI. We also demonstrated sex-specific differences in correlations between BAI and total percentage body fat. In contrast to Bergman et al.'s study [5], in the present study the relationships between percentage fat and BAI for males and females lie on very different linear representations. As expected, males are localized on the lower end of the curve, because males tend to have a lower adiposity compared to females, this is also reflected in a lower value of mean BAI for men.

Although BMI appeared to be equivalently correlated to PBF in both men and women, BAI was more strongly correlated with PBF in men than in women indicating that BAI predicts PBF among males quite well. As BAI is based on hip circumference, the finding is counterintuitive to the general assumption that hip circumference might be more informative compared to waist circumference to predict PBF among women than men [17]. The lower correlations for women may be related to differences in the distribution of fat between sexes and aging—women tend to have a more peripheral than central fat distribution in early adulthood [18], yet gain central fat with increasing age [19]. However, different correlations of BAI for males versus females reflect the well-established difference in body composition between males and females [20].

Though the correlation of BMI with PBF was higher than BAI, sensitivity and specificity of BAI to predict hypertension were better than BMI as well as other anthropometric markers, except for BMI defined by our cut-off [16]. A BMI value of >30 kg/m^2^ has high specificity but poor sensitivity for diagnosing hypertension; as a result, it may miss many patients with true excess adiposity. In addition, WHtR overestimates substantially the prevalence of hypertension in the present population, especially in women. Sensitivity and specificity of WHR were found to be equally poor in both sexes [16].

In Bergman's study, percentage fat was positively correlated with hip circumference and negatively correlated with height; however, this might not be the case in other populations. In the present study, although the correlation of PBF with hip circumference was stronger than weight, the correlation with height was not negative (not shown in data). Furthermore although measuring hip circumference is easier in difficult terrain, calculation of BAI is comparatively tedious in the field.

We acknowledge potential limitations inherent in our study. There were a relatively small number of subjects included in the study; therefore, the findings may not be generalizable to other populations, and our analyses may have been underpowered. Further absence of direct measurement of PBF by refined instrument such as DEXA and Bioelectrical Impedance (BI) could also limit our findings. In conclusion, although the correlation of BMI is better than BAI, the sensitivity and specificity of BAI are similar to, if not better than, BMI. BAI can be used as an additional marker in addition to BMI for screening population; however its validity needs to be demonstrated on other populations too, before accepting it as a new marker for body fat measurement to predict cardiovascular and other health risks.

## Figures and Tables

**Table 1:** Mean and standard deviation of BAI, BMI, and PBF and the correlations between these variables.

	Mean (SD)			*r* (95% CI)^1^		
	BAI	BMI	PBF	BAI and PBF	BMI and PBF	*P* value^2^
Men	27.6 (3.8)	26.1 (4.3)	29.0 (5.9)	0.66	0.83	4.36 (0.001)
Women	39.2 (6.4)	29.4 (4.8)	40.7 (3.7)	0.58	0.71	2.68 (0.05)

Total	33.8 (7.9)	27.9 (4.8)	35.8 (6.6)	0.80	0.70	4.02 (0.001)
				3.52 (0.001)^3^	1.66 (0.09)^3^	

^1^All correlations are statistically significant at *P* < 0.001.

^2^Comparing the correlations of BMI and BAI with Fat%.

^3^Comparing the correlations of BMI and BAI with Fat% across gender.

BAI: body adiposity index; BMI: body mass index; PBF: percentage body fat.

**Table 2:** The areas under ROC curve (AUC), optimal cut-off values, and sensitivities and specificities of BAI associated with BMI and hypertension.

	BMI				BP			
	AUC (95% CI)^1^	Cut-off	Sens (%)	Spec (%)	AUC (95% CI)^1^	Cut-off	Sens (%)	Spec (%)
Men								
By our own criteria	0.93 (0.90, 0.96)	25.6	88.4	92.6	0.70 (0.62–0.77)	25.3	83.7	54.0
By WHO criteria	0.90 (0.86, 0.94)	27.2	77.6	91.7				
Women								
By our own criteria	0.93 (0.92, 0.97)	37.7	91.2	85.5	0.62 (0.55–0.68)	37.7	91.2	85.5
By WHO criteria	0.94 (0.90, 0.96)	36.4	74.3	100.0				

^1^All values are statistically significant at *P* < 0.001.

BAI: body adiposity index; BMI: body mass index; BP: blood pressure; AUC: area under curve; CI: confidence interval; Sens: sensitivity; Spec: specificity; WHO: World Health Organization.

**Table 3:** Diagnostic accuracy of BAI to detect blood pressure by different cut-off points.

	AUC	Sensitivity (%)	Specificity (%)	Positive PV	Negative PV
Men					
BAI (optimal-25.6)	0.70	79.47	56.58	82.06	53.75
BAI (WHO-27.2)		57.89	72.37	83.97	40.74
Women					
BAI (optimal-37.7)	0.62	67.68	56.34	64.16	60.15
BAI (WHO-36.4)		71.95	47.88	61.96	59.65

BAI: body adiposity index; AUC: area under curve; PV: positive predictive; WHO: world health organization.

**Table 4:** Odds ratios of hypertension in obese subjects determined by the cut-off points of BAI.

	SBP			DBP			SBP or DBP		
	ORs	95% CI	*P*	ORs	95% CI	*P*	ORs	95% CI	*P*
Women									
BAI (optimal)	2.70	1.70–4.31	0.001	3.94	2.40–6.45	0.001	3.28	1.95–5.51	0.001
BAI (WHO)	2.36	1.47–3.79	0.001	3.39	2.07–5.54	0.001	2.99	1.79–4.99	0.001
Men									
BAI (optimal)	5.32	2.98–9.49	0.001	3.33	1.72–6.63	0.001	4.23	1.85–9.67	0.001
BAI (WHO)	3.60	2.02–6.43	0.001	3.82	1.84–7.90	0.001	4.02	1.56–10.35	0.001
